# Never Repeat the Same Trick Twice—Unless it is Cognitively Impenetrable

**DOI:** 10.1177/2041669518816711

**Published:** 2018-12-26

**Authors:** Vebjørn Ekroll, Evy De Bruyckere, Lotte Vanwezemael, Johan Wagemans

**Affiliations:** Laboratory of Experimental Psychology, University of Leuven (KU Leuven), Belgium; Department of Psychosocial Science, University of Bergen, Norway; Laboratory of Experimental Psychology, University of Leuven (KU Leuven), Belgium; Laboratory of Experimental Psychology, University of Leuven (KU Leuven), Belgium; Laboratory of Experimental Psychology, University of Leuven (KU Leuven), Belgium

**Keywords:** amodal completion, perceptual organization, magic, inattentional blindness, cognitive impenetrability

## Abstract

In their quest for creating magical experiences, magicians rely on a host of psychological factors. Here, we compare tricks based on attentional misdirection with tricks based on amodal completion. Based on the notion that amodal completion is a cognitively impenetrable perceptual phenomenon, we predicted that the tricks based on this perceptual effect should—to a much larger extent than tricks based on attentional misdirection—retain their deceptive power when the tricks are repeated. The results of an experiment with four magic tricks involving attentional misdirection and four magic tricks based on amodal completion lend strong support to this prediction. Asking subjects to try to figure out the secret behind these tricks after one, two, or three presentations of each trick, we found that the observed solution rates for tricks based on attentional misdirection increased much more with repeated viewing than those for tricks based on amodal completion, which remained very low throughout. Thus, the results lend further support to the idea that amodal completion is based on cognitively impenetrable perceptual mechanisms.

## Introduction

Common wisdom among magicians has it that you should never repeat the same trick twice because it makes it easier for the spectators to figure out how it is done. It is fairly obvious that this has to be true: Repeating the trick gives the spectators more time to think about it, and hence, the chances that they are able to figure out the method increase. This, however, is so obvious that it is hardly worth saying. Thus, when magicians point to the dangers of repeating a trick, they probably implicitly refer to something more profound than this trivial point. Obviously, magic tricks tend to be difficult to debunk the first time they are shown, because they were designed by magicians with that aim in mind. Thus, the magicians' warning seems to imply that there is something about magic tricks which makes them easier to debunk the second time around—beyond the trivial influence of more time to think. It is reasonably clear, however, that magic tricks differ with respect to what cognitive and perceptual principles are involved in creating the magical experience (Kuhn, Caffaratti, Teszka, & Rensink, 2014). Thus, it is possible that the warning against repetitions is quite appropriate for some tricks but less so for others. In particular, simple theoretical arguments (to be explained later) suggest that the warning might be appropriate for tricks based on inattentional blindness (Mack & Rock, 1998), but less so for tricks based on cognitively impenetrable perceptual illusions (Ekroll & Wagemans, 2016; Firestone & Scholl, 2015; Kanizsa, 1985; Leslie, 1988; Pylyshyn, 1999).

The reason why repetition can be expected to be risky in the case of tricks based on inattentional blindness is that they entail that a secret move is carried out in full view but not noticed due to a lack of attention. Thus, once the factor that induces the lack of attention—typically the misdirection of attention toward something else—becomes ineffective, the spectator is likely to notice the directly visible secret. The misdirection of attention toward something else is probably less effective when repeated because the spectators are less likely to attend to the things they already have attended to when they view the trick a second time. Hence, although a trick based on attentional misdirection can be very powerful and robust across observers at first presentation, the secret might become less obvious at second presentation. The disappearing cigarette trick investigated by Kuhn and Tatler (2005) is a case in point. Here, the disappearing cigarette is openly dropped into the magician's lap, but spectators typically fail to notice this otherwise obvious event due to attentional misdirection. While the openly visible drop of the cigarette is rarely noticed at first presentation, all of the participants in Kuhn and Tatler's (2005) study noticed it at second presentation.

In the case of tricks based on cognitively impenetrable perceptual illusions, however, it is not obvious why repeating the trick should make it particularly easy for spectators to debunk it. Rather, due to the robust and persistent nature of perceptual illusions, we would expect the spectators to be equally susceptible to it irrespective of the number of presentations. It is, of course, not impossible to realize—on a purely intellectual level—that the trick is based on a perceptual illusion, but this insight does not result from any changes in the (misleading) perceptual experience with repeated presentation. Rather, since the secret to the trick is hidden behind a misleading and persistent perceptual experience, realizing the true secret behind the trick is only possible via abstract logical thinking that questions the veracity of one's own immediate perceptual experience. Given that we (or our perceptual systems) have a strong penchant for imparting reality to our perceptual experiences (Hoffman, Singh, & Prakash, 2015; Jackendoff, 1991; Koenderink, 2011; Leddington, 2016; Mausfeld, 2013; Michotte, 1991; Savardi, Kubovy, & Bianchi, 2012; Vishwanath, 2013), this is not likely to happen very frequently.

**Figure 1.**
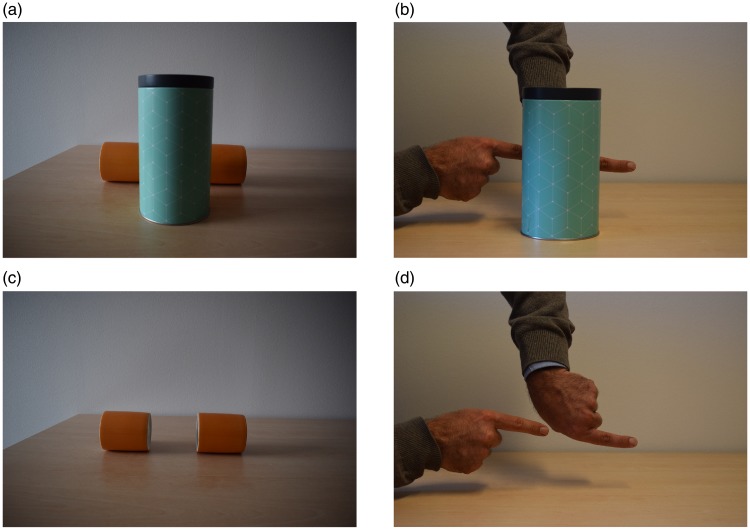
Two demonstrations of amodal completion. (a) One has a curiously compelling and definite impression of a long horizontal cylinder extending unbroken behind the vertical cylinder. Even when one knows that there are just two short cylinders behind the vertical cylinder (c), the impression evoked by viewing panel (a) persists. (b and d) Amodal completion may also evoke perceptual impressions that are impossible in the sense that they are at odds with our general knowledge of objects (Gerbino & Zabai, 2003; Hazenberg & van Lier, 2015).

One might think that these considerations are moot and irrelevant because cognitively impenetrable perceptual illusions only play a marginal role in magic. In a textbook on visual illusions, for instance, Luckiesh (1922) claimed that “there are fewer illusions found in the practice of the magician than is generally supposed,” arguing that “the eye usually delivers correctly to the intellect, but the judgement errs for various reason” (p. 205). Contrary to this, however, Ekroll and Wagemans (2016) have argued that cognitively impenetrable perceptual illusions play a central and pervasive role in magic. The reason why perceptual illusions in magic are easily overlooked, they argued, is that those most often used in magic involve achievements of the visual system that are so staggering that is difficult to envision that they really are perceptual illusions rather than more “intelligent” high-level inferences. One of these achievements is the visual perception of hidden things known as amodal completion (see Figure 1; Kanizsa, 1985; Michotte & Burke, 1951; Michotte, Thinès, & Crabbé, 1964; van Lier & Gerbino, 2015). As discussed in Ekroll, Sayim, and Wagemans (2017), amodal completion can be thought of as a cognitively impenetrable visual illusion that plays a pivotal and pervasive role in magic.

The aim of the present experiment was to test whether it is indeed true that magic tricks based on a perceptual illusion like amodal completion are more difficult to debunk after repeated presentations than tricks based on other factors such as attention. To anticipate, the results support this hypothesis.

## Method

To assess how robust different kinds of magic tricks are to repetition, we asked 40 participants to try to figure out how eight different magic tricks work (see Table 1 and Supplementary Movies). We used four tricks that are predominantly based on amodal completion (tricks Amodal 1–4) and four tricks that instead involved other kinds of misdirection (mainly attentional misdirection, tricks Attention 1–4). The tricks were performed by a skilled amateur magician. A video clip of each trick was presented 3 times, and the participants orally reported what they thought could be the explanation of the trick after each presentation. These oral reports were recorded on audio for later analysis. After the first presentation of each trick, the participants were also asked (a) whether they already knew how the trick worked before they saw it and (b) to rate how magical they found it on a scale from 1 (*not magical at all*) to 10 (*very magical*). Since we were particularly interested in comparing the tricks based on amodal completion with the tricks involving attentional misdirection, we balanced the order of presentation across two groups of participants such that half of the participants viewed the tricks in the sequence Amodal 1, Attention 1, Amodal 2, Attention 2, and so forth, and the other half viewed them in the sequence Attention 1, Amodal 1, Attention 2, Amodal 2, and so forth.

**Table 1.** Descriptions of the Magic Tricks Used in the Experiment.

Trick label	Trick name	Spectator experience	Explanation	Underlying perceptual or cognitive principles (tentative)
Amodal 1	Spoon bending	A spoon is bending.	The spoon is already bent before the trick, but aligning its head with a spare handle creates the illusion of a straight spoon (see Figure 3 in Ekroll et al., 2017).	Amodal completion
Amodal 2	Cut and restored rope	A rope is cut in the middle and then shown to be unsevered.	The magician cuts through a loop in the rope, which seems to be located in the middle of the rope, but actually only a small piece is being cut off. Furthermore, the ends of the long part and the short part of the severed rope are hidden in the magician's hand (see Barnhart, 2010).	Amodal completion (operating twice, creating false correspondences between parts of the rope(s) in two different stages of the trick)
Amodal 3	Knife through arm	The magician cuts through his arm with a knife. When the knife is removed, the arm is undamaged.	The knife has an opening in the blade with space for the arm (see Figure 5 in Ekroll et al., 2017).	Amodal completion in combination with factors making the tendency to completion of the knife stronger than the tendency to completion of the arm (Gerbino & Zabai, 2003; Vrins, de Wit, & van Lier, 2009)
Amodal 4	Penetrating dollar bills	A dollar bill penetrates another dollar bill twice, but both bills are shown to be complete.	A fold in one of the bills is used to create the illusion that one bill is passing through in front of one part of the other bill, but it is really passing through in the back.	Amodal completion
Attention 1	Strike vanish (Kaufman, 1989)	A coin lies in the open hand of the magician. The magician taps the hand three times with a pencil. On the third tap, the coin has disappeared.	The coin is being thrown into the other hand just before the third tap.	Temporal modulation of attention based on rhythm (Barnhart, Ehlert, Goldinger, & Mackey, 2018)—Reduced visibility due to speed (Hergovich, Gröbl, & Carbon, 2011)
Attention 2	Coin through table (Ganson, 1958)	The magician holds a coin in his hand, hits the table with the hand and the coin has somehow disappeared.	The magician drops the coin into his lap just before hitting the table.	Attentional misdirection or misperception of intentions (Macknik et al., 2008; Scholl & Gao, 2013; Van de Cruys, Wagemans, & Ekroll, 2015)
Attention 3	Vanishing ball (Kuhn & Land, 2006; Triplet, 1900)	The magician throws a ball in the air three times. The third time, the ball seems to have disappeared.	The magician only pretends to throw the ball and keeps it palmed in his hand.	Expectations or predictions based on long-term knowledge of kinematics (Kuhn & Rensink, 2016; Thomas & Didierjean, 2016)—Modal completion in time (Beth & Ekroll, 2015)
Attention 4	Vanishing cigarette	The magician tries to light his cigarette, but it disappears (Kuhn & Tatler, 2005).	The cigarette is openly dropped into the magician's lap.	Inattentional blindness (Kuhn & Tatler, 2011)

### Participants and Ethical Approval

Forty acquaintances of the researchers participated in the experiment. Sixty percent of the participants were female and the average age was 30.9 years (range: 21–72). One of the participants declared to perform magic as an amateur, the others indicated that they did not perform magic at all. All methods and procedures were approved by the Ethical Committee of the Faculty of Psychology and Educational Sciences at KU Leuven, and written informed consent was obtained prior to the experiments.

## Results

The percentage of subjects who indicated that they knew a given trick in advance was generally low, at most 10% (see Table 2). Where a subject knew the trick in advance, the corresponding ratings were excluded in all further analysis.

**Table 2.** Number of Subjects Who Knew the Tricks in Advance (Out of 40).

Trick type	Amodal completion				Attention			
Trick number	1	2	3	4	1	2	3	4
*N*	1	4	3	2	2	0	4	0
%	2.5	10	7.5	5	5	0	10	0

The solutions offered by the participants were initially coded into five categories. In addition to the categories where “complete true solutions” or “no possible solutions” were given, we also used categories for “other plausible solutions,” “partial true solutions,” and “partial other plausible solutions.” The coding was performed by the authors E. D. B. and L. V. in working together as well as by a naïve student research assistant who worked independently and was unaware of the hypotheses. Comparison of the two sets of codings revealed quite some disagreement across some of the categories, but the category “complete true solution” was distinguished from the other categories with a high degree of consistency (96.7%). We therefore only analyzed the data in terms of complete true solutions. The analyses presented are based on the codings of the authors E. D. B. and L. V. Essentially identical results were obtained using the codings of the naïve research assistant.

**Figure 2.**
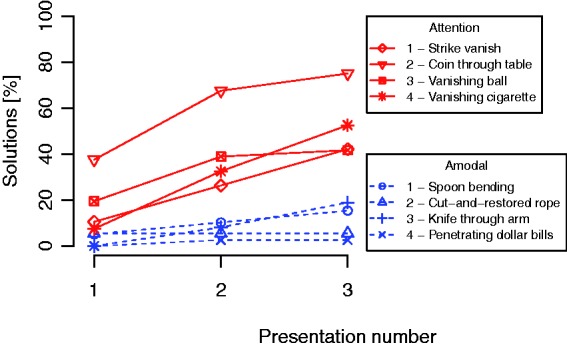
Percentage of the subjects who found out the secret behind the magic tricks plotted against the number of times the trick had been viewed. Note that the solution rates are always lower for the tricks based on amodal completion (dotted lines) and tend to increase less with the number of presentations.

It sometimes happened that a subject mentioned the right solution to a trick at an early presentation, but nevertheless offered another, false solution at a later presentation. In these cases, we regarded the trick as solved. Figure 2 shows the percentage of subjects who mentioned the correct secret behind each trick plotted against the presentation number. As can be seen, the solution rates for the tricks based on amodal completion are low and do not increase very much with repeated presentations. The corresponding solution rates for the other tricks are always larger than for any of the tricks based on amodal completion and tend to increase more with repeated presentations.

To evaluate the strength of the statistical evidence for or against differences between the two types of tricks, we computed Bayes factors using the “contingencyTableBF” function of the *BayesFactor package for R* (Morey & Rouder, 2015). Generally, a Bayes-factor of 3 or more can be considered as evidence in favor of a difference, a Bayes factor of 1/3 or less can be considered as evidence against a difference, and a Bayes factor between 1/3 and 3 can be taken to indicate that the evidence is inconclusive (Dienes, 2008). Comparing the overall proportion of solutions for the two types of trick at the first presentation, we obtain a Bayes factor of 56664. This means that the data are 56664 times more likely given the alternative hypothesis than given the null hypothesis. According to Jeffreys' (1948) terminology, this can be considered “decisive evidence” for a difference. The changes after repeated presentations are best considered in terms of the incidence of solutions, that is, the proportion of solutions among those observers who had not already solved the trick at the previous presentation. Comparing the incidence of solutions for the two types of tricks at the second presentation (i.e., the proportion of solutions among the participants who had not already solved the trick at the first presentation), we obtain a Bayes factor of 687146 (also “decisive evidence” for a difference). The same comparison at the third presentation yields a Bayes factor of 113 (also “decisive evidence” for a difference).

A problem with this analysis is that the higher incidence of solutions at repeated presentations for the tricks based on attentional misdirection may, at least to some extent, be a consequence of the fact that these tricks are easier to solve to begin with.

Thus, an entirely unbiased analysis of the differences in the effect of repetition *per se* presupposes tricks that are equally difficult to solve to begin with. Fortunately, a subset of the tricks including two tricks based on amodal completion (Amodal 1—spoon bending and Amodal 2—cut-and-restored rope) and two tricks based on attentional misdirection (Attention 1—strike vanish and Attention 4—vanishing cigarette) have very similar initial solution rates (Amodal 1 = 5% and Amodal 2 = 6%, Attention 1 = 10% and Attention 4 = 8%). Restricted to those four tricks, we obtain a Bayes factor of 0.15, which can be interpreted as “substantial evidence” against any difference in the initial solution rates for the tricks based on amodal completion and those based on attentional misdirection. Comparing the incidences at the second presentation, however, we obtain a Bayes factor of 55, which constitutes “very strong evidence” for a difference between the two types of tricks, and for the third presentation, we obtain a Bayes factor of 170, which constitutes “decisive evidence.”

**Figure 3.**
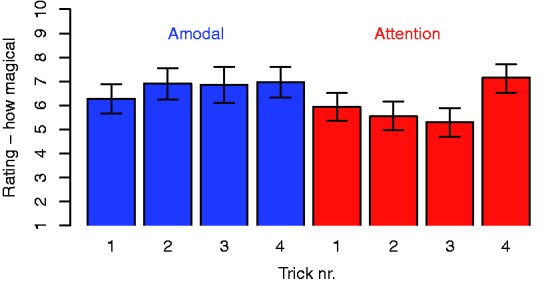
Average ratings of how magical the subjects experienced the different tricks to be, on a scale from 1 (*not magical at all*) to 10 (*very magical*). The error bars show 95% confidence intervals obtained with bootstrapping.

Figure 3 shows the subjects' average ratings of how magical they found the different tricks to be. The average rating across the tricks based on amodal completion (6.70) is somewhat higher than the corresponding value for the other tricks (5.93). The difference between the two distributions of averaged values is statistically significant according to a Wilcoxon test (*p* = .001). It is worth noticing, though, that the ratings of trick Attention 4 (vanishing cigarette) are comparable to the average ratings of the tricks based on amodal completion.

**Figure 4.**
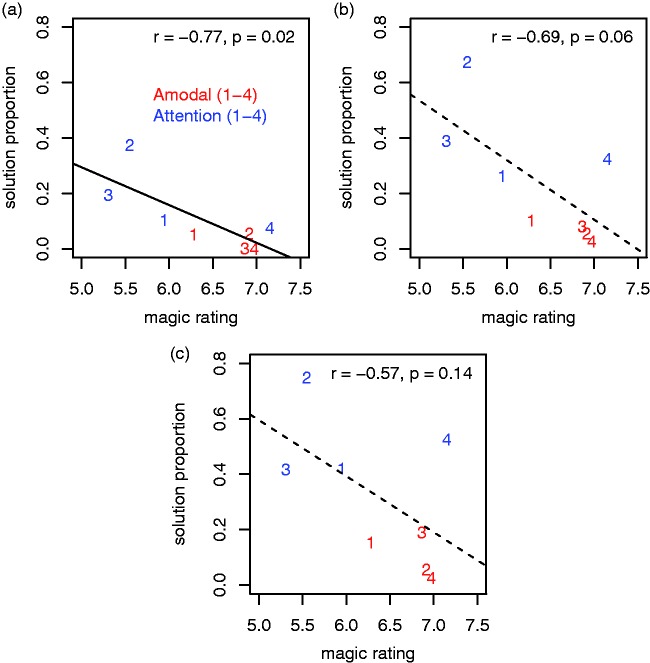
(a) Proportion of subjects who found the secret behind the tricks after the first presentation plotted against the corresponding magic ratings. (b and c) Corresponding plots for the second and third presentation of the tricks. The linear regression lines have a negative slope in all three cases, but they are dashed in panels (b) and (c) to indicate that the correlation failed to be significant at the 5% level in these cases.

Figure 4 shows the proportion of correct solutions for each of the tricks plotted against the corresponding average magic ratings, shown separately for each of the three presentation times. Unsurprisingly, the average magic ratings for the tricks correlate negatively with the proportions of solutions at all presentation times (*r*_1_ = −.77, *r*_2_ = −.69, and *r*_3_ = −.57), but the correlation is only statistically significant at the 5% level for solution rates after the first presentation (*p*_1_ = .02, *p*_2_ = .06, and *p*_3_ = .14). Given that the magic ratings were only collected after the first presentation of each trick, it is perhaps not so surprising that the relationship between the magic ratings and the solution ratings becomes weaker at later presentations. In terms of the coefficients of determination ($r_{1}^{2}$ = .60, $r_{2}^{2}$ = .47, and $r_{2}^{3}$ = .32), the relationship is about halved after the third presentation.

## Discussion

The aim of this experiment was to test the hypothesis that magic tricks based on amodal completion (Ekroll et al., 2017) are more robust than tricks based on other factors such as attentional misdirection. A clear result of our experiment is that solution rates for the tricks based on amodal completion are very low after the first presentation and increase only marginally with repeated presentations (Figure 2). Even after the third presentation, the *highest* solution rate for any of the tricks based on amodal completion was only 19%, while the *lowest* solution rate for any of the other tricks was more than twice of that (42%). Compared with all of the other tricks, all of the tricks based on amodal completion have lower solution rates at all presentation times. In principle, one may argue that the larger increases in solution rates with repeated presentation for the tricks based on attentional misdirection may, at least in part, be a consequence of the higher initial solution rates for these tricks. However, as we have shown, some of the tricks based on attentional misdirection have initial solution rates which are almost as low as those of the tricks based on amodal completion, but the solution rates nevertheless increase much more quickly with repetition. Thus, we can be fairly confident that tricks based on amodal completion are much less susceptible to the detrimental effects of repetition than the tricks based on attentional misdirection.

In principle, the lower initial solution rates observed for the tricks based on amodal completion could result from an unbalanced sampling of tricks: Perhaps the attentional misdirection tricks we selected just happened to be of limited quality, while the amodal completion tricks happened to be of higher quality. Relatedly, it is probably fair to say that performing the tricks involving attentional misdirection require considerably more skill on the part of the magician than performing the tricks based on amodal completion, which are almost self-working. Thus, any unintended glitches in the performance may be expected to have a more detrimental effect on the tricks involving attentional misdirection. Consistent with this kind of argument, the magic ratings were somewhat lower for the tricks involving attentional misdirection than for the tricks based on amodal completion (Figure 3). Thus, our experiment does not provide strong evidence that tricks based on attentional misdirection are generally inferior to tricks based on amodal completion with respect to their deceptive power at the first presentation. As we have already argued earlier, though, our results strongly suggest that tricks based on amodal completion are considerably more robust at repeated presentations.

**Figure 5.**
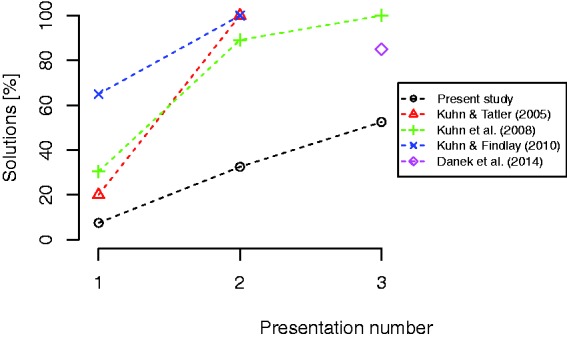
Comparison of results from different studies investigating the vanishing cigarette trick. Although there are differences between the studies, the overall pattern is that the solution rates increase markedly with repeated presentations. From the studies where more than one condition was investigated, we plotted the data from the condition which was most similar to the conditions of the present experiment.

Some of the tricks used in the present experiment have been investigated previously. In Figure 5, results from several studies investigating the vanishing cigarette trick (Danek, Fraps, von Müller, Grothe, & Öllinger, 2014; Kuhn & Findlay, 2010; Kuhn & Tatler, 2005; Kuhn, Tatler, Findlay, & Cole, 2008) are plotted along with the corresponding results from our study. Although there are differences between the results from the different studies, the overall pattern of results is that the solution rates increase markedly with repeated presentations. Several factors may have contributed to the differences between the studies, such as whether the trick was presented live or on video (Kuhn et al., 2008), the details of the criterion used, the timing of the drop, the visual salience of the cigarette, or the quality of the misdirection. In our study, the magician performing the tricks was aware of the hypothesis, and a subtle experimenter effect (Rosenthal, 1966), where this knowledge may have unconsciously influenced the quality of the performance of the different tricks cannot be ruled out. It is clear, though, that this can only explain overall differences in the solution rates, not differences in the changes with repeated viewing.

Danek et al. (2014) also investigated a cut-and-restored rope trick (trick number 4 in their study), which we presume is similar or identical to the one used in our study (Amodal 2). They reported a solution rate of 25% after three presentations, which is somewhat higher than the corresponding value obtained in our study (6%). Relatively to the solution rates for the other 33 tricks investigated in their study, though, the 25% solution rate is still quite low—only three of them had a lower solution rate. Thus, both our and their results agree in suggesting that tricks based on amodal completion are particularly robust to repeated presentations.

On the whole, the present findings clearly demonstrate that tricks based on amodal completion are very difficult to debunk, even after repeated presentations. This is exactly what you would expect for tricks that are based on a cognitively impenetrable perceptual illusion (Ekroll & Wagemans, 2016). First, it should be particularly difficult to even imagine the secret behind a trick based on a perceptual illusion, because it requires you to question the veracity of your immediate perceptual experience. Second, the well-established robustness and persistence of perceptual illusions (Firestone & Scholl, 2015; Leslie, 1988; Pylyshyn, 1999) imply that magic tricks based on them should be correspondingly robust to repetition. Thus, the present findings add further support to the notion that amodal completion is a genuine product of perceptual mechanisms rather than just visual imagery or cognitive guesswork (Ekroll, Sayim, & Wagemans, 2013; Ekroll, Sayim, Van der Hallen, & Wagemans, 2016; Ekroll et al., 2017; Ekroll & Wagemans, 2016; Kanizsa, 1985; Kanizsa & Gerbino, 1982; Michotte et al., 1964).

On a general level, one would expect that not only tricks based on amodal completion but also tricks based on any other perceptual illusion should be resistant to repeated viewing (Ekroll & Wagemans, 2016). Interestingly, Cui, Otero-Millan, Macknik, King, and Martinez-Conde (2011) found that a trick involving simulated coin tosses is highly resistant to repetition, and that counter to common wisdom, the trick is not driven by gaze-related social misdirection cues. A potential interpretation of this finding is that the trick is actually driven by a perceptual illusion where the illusory motion path is a result of spatiotemporal modal completion (Beth & Ekroll, 2015).

One might also argue that if a certain magic trick is very resistant to repeated viewing, this suggests that it may rely on a perceptual illusion. Thus, studying magic and identifying tricks that have this property may provide new hypotheses and perspectives that challenge traditional notions about what should count as a perceptual illusion (Ekroll & Wagemans, 2016).

## Supplementary Material

Supplementary material

Supplementary material

Supplementary material

Supplementary material

Supplementary material

Supplementary material

Supplementary material

Supplementary material

## References

[bibr1-2041669518816711] Barnhart A. S. (2010) The exploitation of Gestalt principles by magicians. Perception 39: 1286–1289. 21125955 10.1068/p6766

[bibr2-2041669518816711] Barnhart A. S. Ehlert M. J. Goldinger S. D. Mackey A. D. (2018) Cross-modal attentional entrainment: Insights from magicians. Attention, Perception, & Psychophysics 80: 1240–1249. doi:10.3758/s13414-018-1497-8. 10.3758/s13414-018-1497-8 PMC6035090 29520711

[bibr3-2041669518816711] Beth T. Ekroll V. (2015) The curious influence of timing on the magical experience evoked by conjuring tricks involving false transfer: Decay of amodal object permanence?. Psychological Research 79: 513–522. 24941913 10.1007/s00426-014-0584-2

[bibr4-2041669518816711] Cui J. Otero-Millan J. Macknik S. L. King M. Martinez-Conde S. (2011) Social misdirection fails to enhance a magic illusion. Frontiers in Human Neuroscience 5: 103 doi: 10.3389/fnhum.2011.00103. 22046155 10.3389/fnhum.2011.00103 PMC3202226

[bibr5-2041669518816711] Danek A. H. Fraps T. von Müller A. Grothe B. Öllinger M. (2014) Working wonders? Investigating insight with magic tricks. Cognition 130: 174–185. 24300080 10.1016/j.cognition.2013.11.003

[bibr6-2041669518816711] Dienes Z. (2008) Understanding psychology as a science: An introduction to scientific and statistical inference, New York, NY: Macmillan International Higher Education.

[bibr7-2041669518816711] Ekroll V. Sayim B. Wagemans J. (2013) Against better knowledge: The magical force of amodal volume completion. i-Perception 4: 511–515. 25165509 10.1068/i0622sas PMC4129385

[bibr8-2041669518816711] Ekroll V. Sayim B. Van der Hallen R. Wagemans J. (2016) Illusory visual completion of an object's invisible backside can make your finger feel shorter. Current Biology 26: 1029–1033. 27040774 10.1016/j.cub.2016.02.001

[bibr9-2041669518816711] Ekroll V. Sayim B. Wagemans J. (2017) The other side of magic: The psychology of perceiving hidden things. Perspectives on Psychological Science 12: 91–106. 28073329 10.1177/1745691616654676

[bibr10-2041669518816711] Ekroll V. Wagemans J. (2016) Conjuring deceptions: Fooling the eye or fooling the mind?. Trends in Cognitive Sciences 20: 486–489. 27212588 10.1016/j.tics.2016.04.006

[bibr11-2041669518816711] Firestone C. Scholl B. (2015) Cognition does not affect perception: Evaluating the evidence for ‘top-down’ effects. Behavioral and Brain Sciences 20: 1–77. 10.1017/S0140525X15000965 26189677

[bibr12-2041669518816711] Ganson, L. (1958). *The magic of Slydini*. London: Harry Stanley, Unique Magic Studios.

[bibr13-2041669518816711] Gerbino W. Zabai C. (2003) The joint. Acta Psychologica 114: 331–353. 14670703 10.1016/j.actpsy.2003.10.002

[bibr14-2041669518816711] Hazenberg S. J. van Lier R. (2016) Disentangling effects of structure and knowledge in perceiving partly occluded shapes: An ERP study. Vision Research 126: 109–119. 26475087 10.1016/j.visres.2015.10.004

[bibr15-2041669518816711] Hergovich A. Gröbl K. Carbon C. C. (2011) The paddle move commonly used in magic tricks as a means for analysing the perceptual limits of combined motion trajectories. Perception 40: 358–366. 21692425 10.1068/p6866

[bibr16-2041669518816711] Hoffman D. D. Singh M. Prakash C. (2015) The interface theory of perception. Psychonomic Bulletin & Review 22: 1480–1506. 26384988 10.3758/s13423-015-0890-8

[bibr17-2041669518816711] Jackendoff R. (1991) The problem of reality. Noûs 25: 411–433.

[bibr18-2041669518816711] Jeffreys H. (1948) Theory of probability, Oxford, England: Oxford University Press/Clarendon Press.

[bibr19-2041669518816711] Kanizsa G. (1985) Seeing and thinking. Acta Psychologica 59: 23–33. 4024981 10.1016/0001-6918(85)90040-x

[bibr20-2041669518816711] Kanizsa G. Gerbino W. (1982) Amodal completion: Seeing or thinking?. In: Beck J. (eds) Organization and representation in perception, Hillsdale, NJ: Erlbaum, pp. 167–190.

[bibr21-2041669518816711] Kaufman, R. (1989). *Williamson's wonders*, New York, NY: Kaufman and Greenberg.

[bibr22-2041669518816711] Koenderink, J. (2011). Vision as a user interface. In *IS&T/SPIE electronic imaging* (pp. 786504–786504). Bellingham, WA: International Society for Optics and Photonics.

[bibr23-2041669518816711] Kuhn G. Caffaratti H. Teszka R. Rensink R. (2014) A psychologically-based taxonomy of misdirection. Frontiers in Psychology 5: 1392. 25538648 10.3389/fpsyg.2014.01392 PMC4260479

[bibr24-2041669518816711] Kuhn G. Findlay J. M. (2010) Misdirection, attention and awareness: Inattentional blindness reveals temporal relationship between eye movements and visual awareness. Quarterly Journal of Experimental Psychology 63: 136–146. 10.1080/17470210902846757 19459083

[bibr100-2041669518816711] Kuhn, G., & Land, M. F. (2006). There's more to magic than meets the eye. *Current Biology*, *16*(22), R950–R951. 10.1016/j.cub.2006.10.012 17113372

[bibr25-2041669518816711] Kuhn G. Tatler B. W. (2005) Magic and fixation: Now you don't see it, now you do. Perception 34: 1155–1161. 16245492 10.1068/p3409bn1

[bibr26-2041669518816711] Kuhn G. Tatler B. W. (2011) Misdirected by the gap: The relationship between inattentional blindness and attentional misdirection. Consciousness and Cognition 20: 432–436. 20943415 10.1016/j.concog.2010.09.013

[bibr27-2041669518816711] Kuhn G. Tatler B. W. Findlay J. M. Cole G. G. (2008) Misdirection in magic: Implications for the relationship between eye gaze and attention. Visual Cognition 16: 391–405.

[bibr28-2041669518816711] Kuhn G. Rensink R. A. (2016) The vanishing ball illusion: A new perspective on the perception of dynamic events. Cognition 148: 64–70. 26735583 10.1016/j.cognition.2015.12.003

[bibr29-2041669518816711] Leddington J. (2016) The experience of magic. The Journal of Aesthetics and Art Criticism 74: 253–264.

[bibr30-2041669518816711] Leslie A. M. (1988) The necessity of illusion: Perception and thought in infancy. In: L. Weiskrantz (eds) Thought without language, Oxford, England: Clarendon Press, pp. 185–210.

[bibr31-2041669518816711] Luckiesh M. (1922) Visual illusions, New York, NY: Dover.

[bibr32-2041669518816711] Mack A. Rock I. (1998) Inattentional blindness, Cambridge, MA: MIT press.

[bibr33-2041669518816711] Macknik S. L. King M. Randi J. Robbins A. Teller, Thompson J. Martinez-Conde S. (2008) Attention and awareness in stage magic: Turning tricks into research. Nature Reviews Neuroscience 9: 871–879. 18949833 10.1038/nrn2473

[bibr34-2041669518816711] Mausfeld R. (2013) The attribute of realness and the internal organization of perceptual reality. In: Albertazzi L. (eds) Handbook of Experimental Phenomenology: Visual Perception of Shape, Space and Appearance, Chichester, England: Wiley, pp. 91–118.

[bibr35-2041669518816711] Michotte A. (1991) The real and the unreal in the image. In: Thinès G. Costall A. Butterworth G. (eds) Michotte's experimental phenomenology of perception, Hillsdale, NJ: Erlbaum, pp. 187–197.

[bibr36-2041669518816711] Michotte, A., & Burke, L. (1951). Une nouvelle énigme dans la psychologie de la perception: Le ‘donne'e amodal' dans l'expe'rience sensorielle. [A novel enigma in the psychology of perception: The amodally given in sensory experience]. In *13th International congress of psychology: Proceedings and papers* (pp. 179–180).

[bibr37-2041669518816711] Michotte A. Thinès G. Crabbé G. (1964) Les compléments amodaux des structures perceptives, Louvain, Belgium: Publications Universitaires, Studia Psychologica [Amodal completions of perceptual structures].

[bibr38-2041669518816711] Morey, R. D., & Rouder, J. N. (2015). *BayesFactor: Computation of Bayes Factors for Common Designs*. R package version 0.9.12-2. Retrieved from https://CRAN.R-project.org/package=BayesFactor.

[bibr39-2041669518816711] Pylyshyn Z. (1999) Is vision continuous with cognition? The case for cognitive impenetrability of visual perception. Behavioral and Brain Sciences 22: 341–365. 11301517 10.1017/s0140525x99002022

[bibr40-2041669518816711] Rosenthal R. (1966) Experimenter effects in behavioral research, New York, NY: Appleton-Century-Crofts.

[bibr41-2041669518816711] Savardi U. Kubovy M. Bianchi I. (2012) The genesis of the awareness of illusions. In: Calabi C. (eds) Perceptual illusions, Basingstoke, England: Palgrave McMillan, pp. 75–84.

[bibr42-2041669518816711] Scholl B. J. Gao T. (2013) Perceiving animacy and intentionality: Visual processing or higher-level judgment?. In: Rutherford M. D. Kuhlmeier V. A. (eds) Social perception: Detection and interpretation of animacy, agency, and intention, Cambridge, MA: The MIT Press, pp. 197–230.

[bibr43-2041669518816711] Thomas C. Didierjean A. (2016) No need for a social cue! A masked magician can also trick the audience in the vanishing ball illusion. Attention, Perception, & Psychophysics 78: 21–29. 10.3758/s13414-015-1036-9 26676869

[bibr101-2041669518816711] Triplett, N. (1900). The psychology of conjuring deceptions. *The American Journal of Psychology*, *11*(4), 439–510.

[bibr44-2041669518816711] van Lier R. Gerbino W. (2015) Perceptual completions. In: J. Wagemans (eds) Oxford handbook of perceptual organization, Oxford, England: Oxford University Press, pp. 294–320.

[bibr45-2041669518816711] Van de Cruys S. Wagemans J. Ekroll V. (2015) The put-and-fetch ambiguity: How magicians exploit the principle of exclusive allocation of movements to intentions. i-Perception 6: 86–90. 28299166 10.1068/i0719sas PMC4950023

[bibr46-2041669518816711] Vrins S. de Wit T. C. J. van Lier R. (2009) Bricks, butter, and slices of cucumber: Investigating semantic influences in amodal completion. Perception 38: 17–29. 19323133 10.1068/p6018

[bibr47-2041669518816711] Vishwanath D. (2013) Experimental phenomenology of visual 3D space: Considerations from evolution, perception, and philosophy. In: Albertazzi L. (eds) Handbook of experimental phenomenology: Visual perception of shape, space and appearance, Hoboken, NJ: Wiley-Blackwell, pp. 181–204.

